# Photocatalytic degradation of tetracycline antibiotics by RGO-CdTe composite with enhanced apparent quantum efficiency

**DOI:** 10.1038/s41598-023-46120-0

**Published:** 2023-11-03

**Authors:** Suvendu Ghosh, Koushik Chakraborty, Tanusri Pal, Surajit Ghosh

**Affiliations:** 1 Department of Physics, Vidyasagar University, Midnapore, WB 721102 India; 2 Department of Physics, Midnapore College, Midnapore, WB 721101 India

**Keywords:** Environmental sciences, Chemistry, Materials science, Nanoscience and technology

## Abstract

RGO-CdTe composite was synthesized using a straightforward, easy-to-realize, one-pot solvothermal technique. The synthesized composite was characterized by X-ray diffraction (XRD), transmission electron microscopy (TEM), Brunauer-Emmett-Teller method (BET), Raman spectra, UV-Vis absorption, and photoluminescence measurement. The RGO-CdTe composite exhibited 83.6% photocatalytic degradation efficiency for the aqueous tetracycline (TC) antibiotic solution and the apparent quantum yield (AQY) for the same was as high as 22.29% which is 2.63 times higher than that of CdTe. The scavenger investigation demonstrated that although hole acts as the leading active species, despite that, superoxide and hydroxyl radicals have also played crucial roles. The initial pH-dependent photocatalytic performance was measured. The zeta potential of the composite at different pH values was evaluated to establish the photocatalytic performance of the RGO-CdTe towards TC degradation at different pH. The recycling experiment depicts that only a 10% degradation performance declines after 5 times recycle use of the RGO-CdTe photocatalyst. An efficient photocurrent generation in RGO-CdTe thin film device has also been observed. Our study establishes as-synthesized composite of RGO-CdTe as a highly potential, and stable photocatalyst for the degradation of antibiotics from the polluted aqueous environment with a very good photoinduced charge generation efficiency in its solid phase.

## Introduction

Nowadays the widespread usage of antibiotics for disease prevention and treatment is a problem for the environment^1,2^. Additionally, it can be added to feed as a way to speed up the growth of cattle and poultry^3,4^. Antibiotics enter the environment by domestic animal and human excretions (urine and feces), unprofessional handling and/or disposal of unused drugs, and waste streams from the manufacture of antibiotics^5^. Long-term contact with an environment contaminated with antibiotics can increase the development of antimicrobial resistance that not only raises the danger to people and animals but also has an impact on plant development, enzyme secretion, and chlorophyll synthesis^6^. A very recent study has raised grave concerns about antibiotic residues found in the water of four Indian rivers: Yamuna (New Delhi), Gomti (Lucknow), Zuari (Goa), and Cooum (Chennai), which can have detrimental effects on ecosystems and human health^7–9^. Therefore, it′s crucial to reduce the harmfulness of the antibiotics before releasing them into the aquatic environment.

As a broad-spectrum antibiotic, Tetracycline (TC) is widely used to treat a variety of conditions and is considered the second-most often used antibiotic in human activities and livestock breeding due to its low cost and great efficacy^10^. However, excessive and persistent use of TC pollutes the environment, causing widespread social concern^11^. Several techniques including adsorption^12^, ion exchange^13^, membrane filtration^14^, biological processes^15^, electrolysis^16^, ozonation^17^, advanced oxidation processes^18^, and photocatalysis^19^ process have been implemented for the removal of TC. Among these processes, the advanced oxidation processes, and the photocatalysis process are considered the most effective low-cost, easy-to-achieve, eco-friendly methods. The key factor of the photocatalysis process is the efficient generation of charges like electrons, holes, hydroxyl radicals, and superoxide anion radicals. Again, the generation of hydroxyl radical and superoxide anion radical also depends on the formation of exciton and subsequent dissociation into photo-induced electrons and holes.

Owing to their large specific surface area, as well as outstanding optical, electronic, and mechanical properties, the use of semiconducting nanomaterials has received increasing attention in the removal of organic pollutants^20^. Due to its easily configurable size, extraordinary absorption coefficient, near-ideal bandgap for solar-terrestrial light conversion, excellent light-to-electricity conversion efficiency, and remarkable optical, electrical, and mechanical capabilities, CdTe has emerged as a potential candidate for solar light-responsive optoelectronic and photocatalytic applications^21–26^. In comparison to typical crystalline silicon-based solar cells, thin CdTe semiconductor layers employed in light-harvesting devices are capable of producing power from solar radiation at substantially lower costs. In comparison to other PVs, CdTe PV has various benefits including a higher light-to-power conversion efficiency, a smaller carbon footprint, minimal water use, and a faster payback period for electricity^24–26^. Excitons are produced when CdTe is exposed to light of the appropriate wavelength, and they split into free electrons and holes. However, these photo-generated opposite charges have a quick recombination tendency, which is the main constraint to achieving the higher quantum yield of photocatalytic degradation. A successful strategy to achieve high performance is the formation of a composite with different 2-dimensional (2D) materials which hinders the recombination probability^27–29^. To this end, RGO is one of the best choices for researchers. It has been observed that the photocatalytic efficiency of various photocatalysts increases after the attachment of RGO with the catalysts^30–32^. Different RGO-based composites have been utilized for the removal of TC for aqueous environments. Shen et al. synthesized an RGO-Cu_2_O/Bi_2_O_3_ composite for TC degradation under visible-light irradiation^33^. RGO-La_2_Zr_2_O_7_ photocatalyst was synthesized by sol-gel method for the degradation of TC under visible light by Wang et al.^34^. A 2D/2D rGO-Bi_2_WO_6_ heterostructure photocatalyst was successfully utilized by Li et al. for efficient visible-light-driven degradation of TC^35^. Yang et al. utilized BiVO_4_/FeVO_4_@rGO composite as a 3D/2D/2D heterojunction for the removal of TC from water^36^. RGO/CdIn_2_S_4_/g-C_3_N_4_ ternary hybrid system was synthesized by Xiao et al. for the degradation of tetracycline hydrochloride from an aqueous environment^37^. Motivated by the reports we have synthesized a composite of CdTe with RGO where the nanosheets of RGO act as photo-generated electron trappers and will enhance the reactive species generation ability for the degradation of antibiotics. However, no such result is noticed in the literature where CdTe-based composites were used for antibiotic degradation.

Herein, solution-processable RGO-CdTe composite was synthesized by an easy-to-realize, single-step one-pot solvothermal process. The structural, morphological, and optical characterization of the synthesized composite was investigated. For the First time, the RGO-CdTe composite has been evaluated for visible-light-driven photocatalytic degradation of antibiotics like TC. The apparent quantum yield of CdTe also increased 2.63 times after the formation of the composite with RGO. The scavenger experiment demonstrated that holes, superoxide radicals, and hydroxyl radicals play roles in TC degradation. The initial pH of the solution-dependent photocatalytic degradation of TC was studied and observed that the degradation efficiency increases with the increase of pH and becomes maximum at pH = 7 beyond this efficiency decreases with increased pH. This result was established with the help of the Zeta potential of the RGO-CdTe. The outcomes of the study establish that the RGO-CdTe composite is a promising visible-light-driven stable photocatalyst with high efficiency and AQY for the removal of different organic pollutants from the aquatic environment. The photoinduced charge generation in the RGO-CdTe composite was studied by fabricating a thin film photodetector using RGO-CdTe as an active material.

## Experiment

### Materials

Analytical grade Sigma-Aldrich makes chemicals like graphite powder, ethylene glycol [EG, C_2_H_6_O_2_], ethylene diamine [EN, NH_2_CH_2_CH_2_NH_2_], polyvinylpyrrolidone [PVP], cadmium acetate dihydrate [Cd(CH_3_COO)_2_·2H_2_O], sodium tellurite [Na_2_TeO_3_], Sodium nitrate [NaNO_3_], potassium persulfate [K_2_S_2_O_8_], and phosphorus pentoxide [P_2_O_5_] were used in the present synthesis. Other chemicals such as hydrogen peroxide [H_2_O_2_], potassium permanganate [KMnO_4_], hydrochloric, and sulfuric acid were used here from Merck, India.

### Materials synthesis

**Figure 1:**
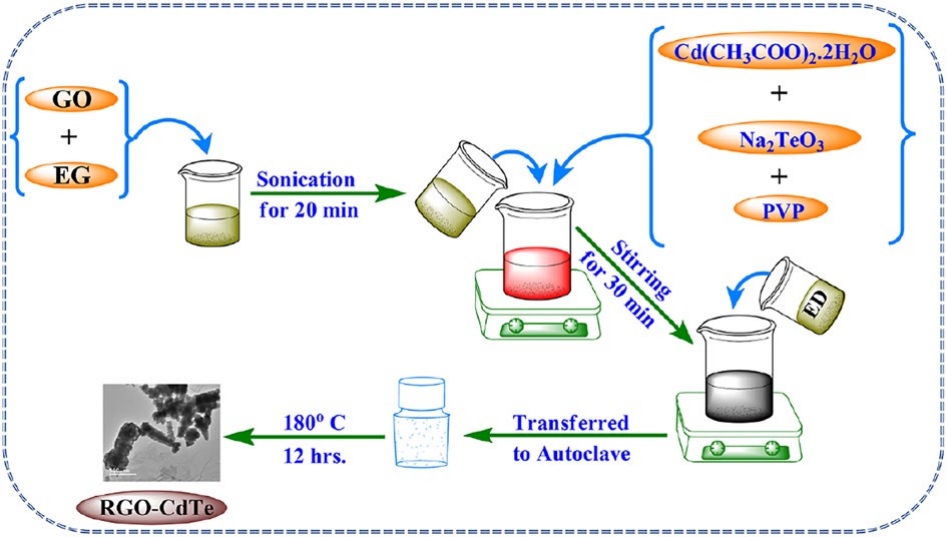
Graphical illustration of RGO-CdTe Composite synthesis.

Graphene oxide (GO) was synthesized by the oxidation of graphite powder. The detailed experimental procedure is mentioned in our earlier report^38^. The RGO-CdTe composites with varying RGO content were synthesized by the cost-effective, easy-to-achieve, single-step, single-pot, solution-processable solvothermal process. Herein, the reduction of GO, CdTe formation, and its attachment on the RGO mat occur concurrently in the in-situ process. In this process, 40 mg of GO was dispersed in 24 ml of EG solvent by 20 min sonication in a water bath and thus a homogeneous solution of GO was obtained. Cd(CH_3_COO)_2_·2H_2_O (1 mM), PVP (0.4 g), and Na_2_TeO_3_ (1 mM) were then poured into the beaker containing GO solution while being stirred magnetically to create a homogenous solution. After 25 min of stirring, 1 ml of ED was added to the solution and continued stirring for the next 5 min. Then, the mixture was transferred into a Teflon-lined stainless-steel autoclave with a 50 ml capacity. The sealed autoclave was transferred to a preheated oven and kept at 180 °C for 12 h. After completion of the reaction the furnace was allowed to cool down naturally to room temperature and a precipitate was formed. The precipitate was collected by centrifugation and washing several times in double distilled water. Then the samples were kept in a vacuum furnace (80 °C) for 5 h. The dried samples were further annealed for 3 h at 200 °C to eradicate the peaks of amine. Thus, the prepared 40 RGO-CdTe composite was named in the present work as RGO-CdTe. Other four different composites 20RGO-CdTe, 30RGO-CdTe, 50RGO-CdTe, and 60RGO-CdTe were synthesized by the same procedure with varying amounts of GO as 20, 30, 50, and 60 mg respectively keeping Cd(CH_3_COO)_2_·2H_2_O amount fixed. The synthesis of CdTe was also carried out using a similar experimental protocol without GO addition. The same solvothermal experimental approach was carried out, while Na_2_TeO_3_, Cd(CH_3_COO)_2_·2H_2_O, and PVP were not added during the synthesis of RGO. The synthesis method of the RGO-CdTe is presented in Fig. 1.

### Materials characterization

The crystallinity and phase formation of the as-synthesized samples were confirmed by a Rigaku-Miniflex X-ray diffractometer with Cu-K_α_ radiation (λ = 0.15418 nm) and worked at 40 kV and 10 mA. The TEM observation was performed with a JEOL-JEM 2100F transmission electron microscope operated at 120 kV. An Autosorb iQ-MP instrument (Quanta chrome instrument) was employed to measure the specific surface area and the pore size by the Brunauer-Emmett-Teller (BET) method using N_2_ adsorption isotherms. The reduction from GO to RGO was confirmed by Raman spectroscopy which was carried out on a micro-Raman spectrometer (Renishaw, inVia Raman Microscope) equipped with a 532 nm excitation laser. An Agilent Cary Series spectrophotometer and a PerkinElmer LS 55 fluorimeter were employed to record the UV-Vis optical absorption and steady-state fluorescence respectively, of the RGO-CdTe composite and CdTe.

### Studies on photocatalytic performance

The performance of CdTe and the RGO-CdTe composite as a photocatalyst was assessed toward the degradation of TC at ambient conditions under a solar light simulator (New port) of illumination intensity 100 mW/cm^2^, with AM 1.5 filter. The intensity of illumination was recorded with an optical power meter (Newport 843-R). In a quartz-made photocatalytic reaction chamber, 30 mL of TC solution (40 mg/L in water) was kept and 30 mg of photocatalyst (CdTe/RGO-CdTe composite) was dispersed on it and used for the photocatalytic study. The reaction chamber was surrounded by a water jacket to avoid any type of heating of the solution during photocatalysis. The solution was kept in the dark for 45 min under stirring to attain an adsorption-desorption equilibrium state. During photocatalysis studies, the temporal absorption spectral changes of 4 ml aqueous TC solution were monitored at an interval of every 5 min.

### Studies on photocurrent generation in thin-film device

The photocurrent generation in RGO-CdTe composite was studied by fabricating a thin film of RGO-CdTe (dispersed in isopropyl alcohol) on a glass substrate by drop casting method. Two parallel electrodes were drawn on the RGO-CdTe thin film by using silver paste. A Kithelay 2611A source meter interfaced with a computer was employed to take the current-voltage data. A Newport solar light simulator was used as the illumination source.

## Results and discussion

**Figure 2:**
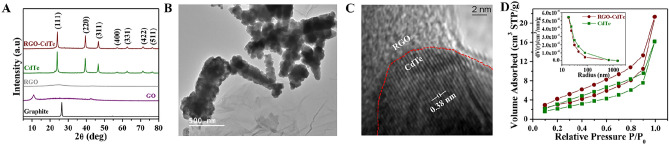
(**A**) Patterns of p-XRD: (top to bottom) RGO-CdTe, CdTe, RGO, GO, and graphite. Image of RGO-CdTe through (**B**) TEM and (**C**) HRTEM (**D**) Nitrogen adsorption-desorption isotherms and the inset is corresponding pore-size distribution curves of as-synthesized CdTe and the RGO-CdTe.

Powder XRD (p-XRD) was employed to evaluate the crystallinity and phase of the synthesized materials. The diffraction pattern of Graphite, GO, RGO, CdTe, and RGO-CdTe composite are compared in Fig. 2A. In the p-XRD of graphite, a distinct sharp peak at 2θ ~ 26.3°, with the corresponding interlayer distance of 0.34 nm is observed. After oxidation, the graphitic peak completely disappears in the diffraction pattern of GO and a peak for the (002) plane, located at 2θ ~ 10.4°, with an inter-planar spacing of 0.85 nm, is clearly observed. The (002) peak of GO completely vanishes in the p-XRD of RGO, whereas a hump appears at 2θ ~ 25°, confirming the graphene restoration after reduction. The indexed peak of CdTe for (111), (220), (311), (400), (331), (422), and (511) fit well with the cubic zinc blende structure [JCPDS Card # 75-2086]^39^. The grain size of CdTe was calculated as 46 nm by the Scherrer equation^40^. The dislocation density (δ) and the microstrain (ε) were estimated using the relations^41^

$$ \delta = \frac{1}{{D^{2} }} \left( {lines/m^{2} } \right)\, and\, \varepsilon = \frac{\beta Cos\theta }{4} $$

as 4.9 × 10^14^ (lines/m^2^) and 8.2 × 10^−2^ respectively. All the CdTe peaks are clearly visible in the p-XRD pattern of the RGO-CdTe composite. Additionally, RGO-CdTe exhibits no peak shift, indicating that the crystallographic phase of CdTe is unaffected by the presence of RGO. Also, in the RGO-CdTe composite′s diffraction profile, the characteristic peak of GO is completely diminished, confirming the reduction of GO and the formation of RGO in the composite. Due to the high crystallinity of CdTe, the signature hump of RGO is suppressed in the p-XRD pattern of the RGO-CdTe composite. The XRD pattern of all the RGO-CdTe composite with varying RGO content is presented in Fig. S1A of Supporting Information (SI). The peak intensity of the XRD pattern of CdTe in the composite decreases with the increase of RGO content in the composite as the CdTe content decreases with the increase of RGO content in the composite.

A representative TEM image of the prepared RGO-CdTe is presented in Fig. 2B, where the folded 2D morphology and the characteristic wrinkle are clearly seen in the RGO substrate. As depicted in Fig. 2B, rod-shaped nanoclusters having an 800 nm average length consisting of nanoparticles of CdTe are dispersed on RGO support. The HRTEM image of the RGO-CdTe composite is presented in Fig. 2C. Clear lattice fringes separated by 0.38 nm, which corresponds to the (111) crystal plane of CdTe, can be observed in the HRTEM image. The RGO/CdTe interface is marked as a red color dotted line. In addition, HRTEM-energy-dispersive X-ray (EDX) also confirmed the presence of C, Cd, and Te elements in the RGO-CdTe composite (Fig. S1B, in SI).

The surface area and porous structural characteristics of CdTe and RGO-CdTe composites are examined using BET surface analysis. Figure 2D depicts the nitrogen adsorption-desorption isotherms for CdTe and RGO-CdTe composites. Both the isotherms exhibit type-IV features^42,43^. The CdTe and the RGO-CdTe composites have corresponding BET surface areas of 8.957 and 11.11 m^2^ g^−1^ respectively. This result demonstrates that the addition of RGO improved the specific surface area of CdTe. In the photocatalytic process, the RGO-CdTe composite can therefore offer more active sites than CdTe. The calculated pore size distribution curves for CdTe and RGO-CdTe composites are displayed in the inset of Fig. 2C. The average pore size for both CdTe and RGO-CdTe composites is almost the same.

**Figure 3:**
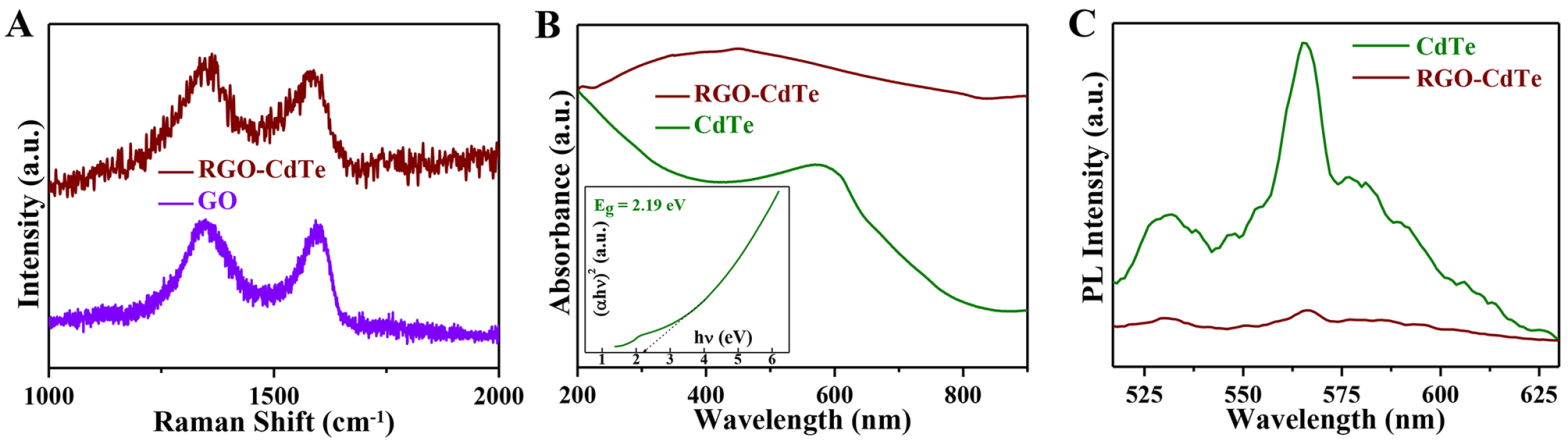
(**A**) Raman spectra: GO and RGO-CdTe composite. (**B**) Optical spectra: CdTe and RGO-CdTe and the inset is plots of energy (*hν*) versus *(αhν)*^2^ of CdTe (**C**) PL spectra: CdTe and RGO-CdTe composite.

The Raman spectra investigation has assessed the reduction of GO and formation of RGO. Figure 3A presents the Raman spectra of GO and RGO-CdTe composite. The D-band centered at 1352 cm^−1^ appears due to the presence of disorderliness in the lattice and the G-band centered at 1599 cm^−1^ appears due to sp^2^-bonded carbon atoms. The D/G intensity ratio of the RGO-CdTe composite increases to 1.24 from 1.07 for GO in Raman spectra, which is a common feature for RGO formation by GO reduction in the composite^42^. In addition to that, the peak position of the D-band remains unaltered. Whereas, as the sp^2^ configuration has been restored in the carbon atoms, the G-band peak position for RGO-CdTe downshifts from 1599 cm^−1^ (for GO) to 1583 cm^−1^^44,45^.

The key factor of a potential photocatalyst is wide-range optical absorption for efficient exciton generation and effectual photo-induced charge transfer to prevent charge recombination. To estimate the optical absorption coefficient and charge transfer, UV-Vis and PL spectroscopy measurements have been carried out and are presented in Fig. 3B,C respectively. The optical absorption spectrum of CdTe depicts an absorption peak at ~ 574 nm (Fig. 3B), whereas the absorption of the composite increases notably in the whole visible region (200–900 nm) after making the composite with RGO. The band gap (E_g_) of the CdTe was estimated from Tauc equation $\alpha h\nu = A\left( {h\nu - E_{g} } \right)^{n}$ where for direct band gap materials the value of n is 1/2, whereas n = 2 and for indirect band gap materials^46,47^. A plot of $\left( {\alpha h\nu } \right)^{2}$ versus $h\nu$ is presented in the inset of Fig. 3B and the bandgap of CdTe was estimated as 2.19 eV. In comparison to bulk CdTe (E_g_ = 1.5 eV), a blue shift in the band gap energy was observed, which might be owing to the quantum confinement effect in CdTe nanoparticles. Due to its larger band gap, CdTe is a promising material for electroluminescent display systems also. The theoretical size dependence band-gap energy can be approximated by the Brus theory^48^ as

$$ E_{g} \left( D \right) = E_{g} \left( {Bulk} \right) + \frac{{h^{2} }}{{2D^{2} }}\left( {\frac{1}{{m_{h}^{*} }} + \frac{1}{{m_{e}^{*} }}} \right) - \frac{{1.8e^{2} }}{\varepsilon D/2}. $$

where $E_{g} \left( {Bulk} \right)$ is the bandgap energy of bulk, *h* is the Plank′s constant, particle size is D, the effective mass of hole and electron are $m_{e}^{*}$ and $m_{h}^{*}$ respectively, ε is the bulk optical dielectric coefficient of CdTe and *e* is the electronic charge. Every material has a diameter where the positive kinetic energy balances the negative Coulombic attraction^49^. Considering the value of $m_{e}^{*} = 0.11m_{0}$^50,51^, where $m_{0}$ is the mass of an electron and ε = 11^52^, $D = 46 nm$ and $E_{g} \left( {Bulk} \right) = 1.5 eV$ gives $E_{g}\left(D \right) = 1.52 \,eV$. The theoretically calculated value of $E_{g} \left( D \right)$ is much lower than our obtained band gap from UV-Vis measurements, this might be due to the large particle size where electron-hole correlation predominates^53^. The optical absorption of all the RGO-CdTe composite with varying RGO content is presented in Fig. S1C of SI. The 40RGO-CdTe composite exhibits the highest absorption in the visible window.

The steady-state PL spectrum of CdTe is shown in Fig. 3C. The emission peak located at 565 nm is assigned to the near band edge emission of CdTe, which is completely quenched in the RGO-CdTe composite. This quenching of luminescence is due to the efficient transfer of photo-induced electrons from the conduction band (CB) of CdTe to the attached RGO sheet, which subsequently reduces the recombination probability of photo-induced electrons and holes inside CdTe^54^. Thus, the RGO-CdTe may be an effective and attractive candidate for photocatalytic applications due to its improved in visible range and effective charge transfer efficiency.

**Figure 4:**
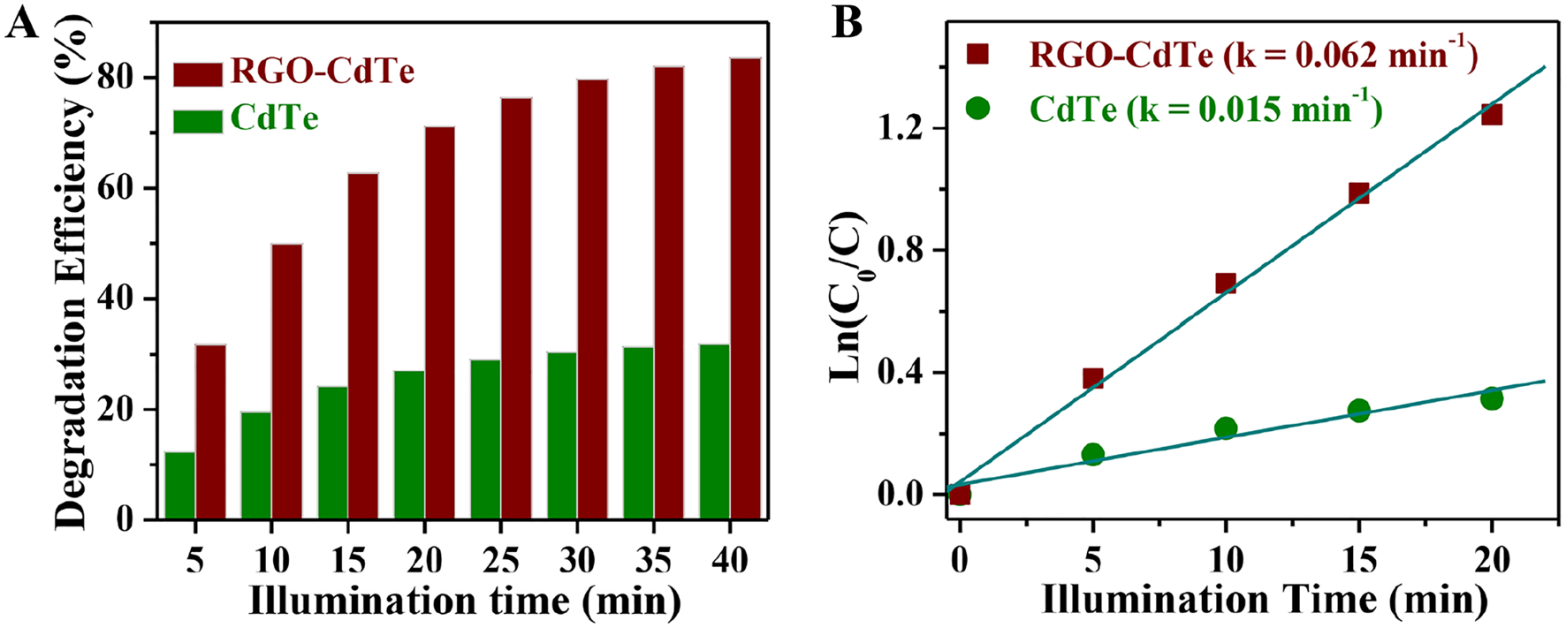
Variation of (**A**) degradation efficiency with the time of illumination and (**B**) Ln (C_0_/C) with the time of illumination for CdTe and RGO-CdTe composite.

The photocatalytic degradation of TC antibiotics in aqueous medium was comparatively studied using CdTe and the RGO-CdTe composite. Optical absorption spectra of the aqueous solution of TC, when RGO-CdTe composite is present under darkness were studied to get a clear picture of the effect of the adsorption process. The temporal absorption spectral changes of TC aqueous solution during the adsorption process with RGO-CdTe composite in darkness are presented in Fig. S2A of SI. As displayed in the figure, 22% of TC was degraded within 45 min, and in the next 40 min, only 3% removal of TC was noted. We also measured the self-degradation of TC under light and a negligible change in TC concentration was observed by the process of self-degradation (Fig. S2B, in SI). These results depict that almost negligible influence of adsorption is present after 45 min. After reaching adsorption-desorption equilibrium (45 min in dark) we shine the light to study the photocatalytic performance of the synthesized catalysts. Figure S3A,B in SI, show the temporal absorption spectral changes of aqueous TC solution during the photodegradation process with CdTe and RGO-CdTe composite, respectively. The degradation efficiency of TC was evaluated as follows^55,56^:

$$ Degradation\, Efficiency\, \left( \% \right) = \left( {1 - \frac{C}{{C_{0} }}} \right) \times 100\% $$

where TC aqueous solution concentrations are C_0_ and C, respectively, at the start of the illumination period (*t* = 0) and at time *t*. The variation of degradation efficiency of CdTe and the RGO-CdTe composite is compared in Fig. 4A. 31.8% TC degradation efficiency was noticed after 40 min of illumination for the CdTe. But in the identical experimental condition when RGO-CdTe composite was used the degradation reached 83.6% for the same period of illumination. The photocatalytic degradation of TC follows a pseudo-first-order kinetic reaction process, and the pseudo-first-order kinetic constant or the degradation rate constant *k* (min^−1^) was calculated by the equation: $Ln \left( {C/C_{0} } \right) = - kt$^57,58^. Figure 4B shows the variation of $Ln \left( {C/C_{0} } \right)$ with the illumination time. It has a linear pseudo-first-order relationship, and k was calculated from the linear fit. Extraordinarily, the value of k for CdTe is 0.015 min^-1^ which increases 4.15 times after making a composite with RGO.

The quantum yield is a crucial parameter for classifying a photocatalyst and determining its efficiency. However, in a dispersed system scattering makes it difficult to accurately count the photons number absorbed by a photocatalyst. To avoid this difficulty, we have calculated the Apparent Quantum Yield (*AQY*) instead of the quantum yield for CdTe and RGO-CdTe for the degradation of TC antibiotics. The *AQY* was calculated as follows^59,60^;

$$ AQY = \frac{Number\, of\, degraded\, molecule}{{Number\, of\, incident\, photon}} \times 100 \% $$

where the number of photons was calculated from the total energy incident on the solution divided by the energy of each photon, both for the particular wavelength corresponding to the band gap of the catalyst. The total incident energy was calculated by measuring the intensity of incident light in the unit of Wm^−2^ multiplied by the exposed area. The detailed calculation of *AQY* is presented in the SI. The estimated *AQY* of the RGO-CdTe composite is 22.29%, which is 2.63 times higher than CdTe after 40 min of Xenon lamp illumination. The increase of *AQY* is due to the prevention of electron-hole recombination in the RGO-CdTe composite.

**Figure 5:**
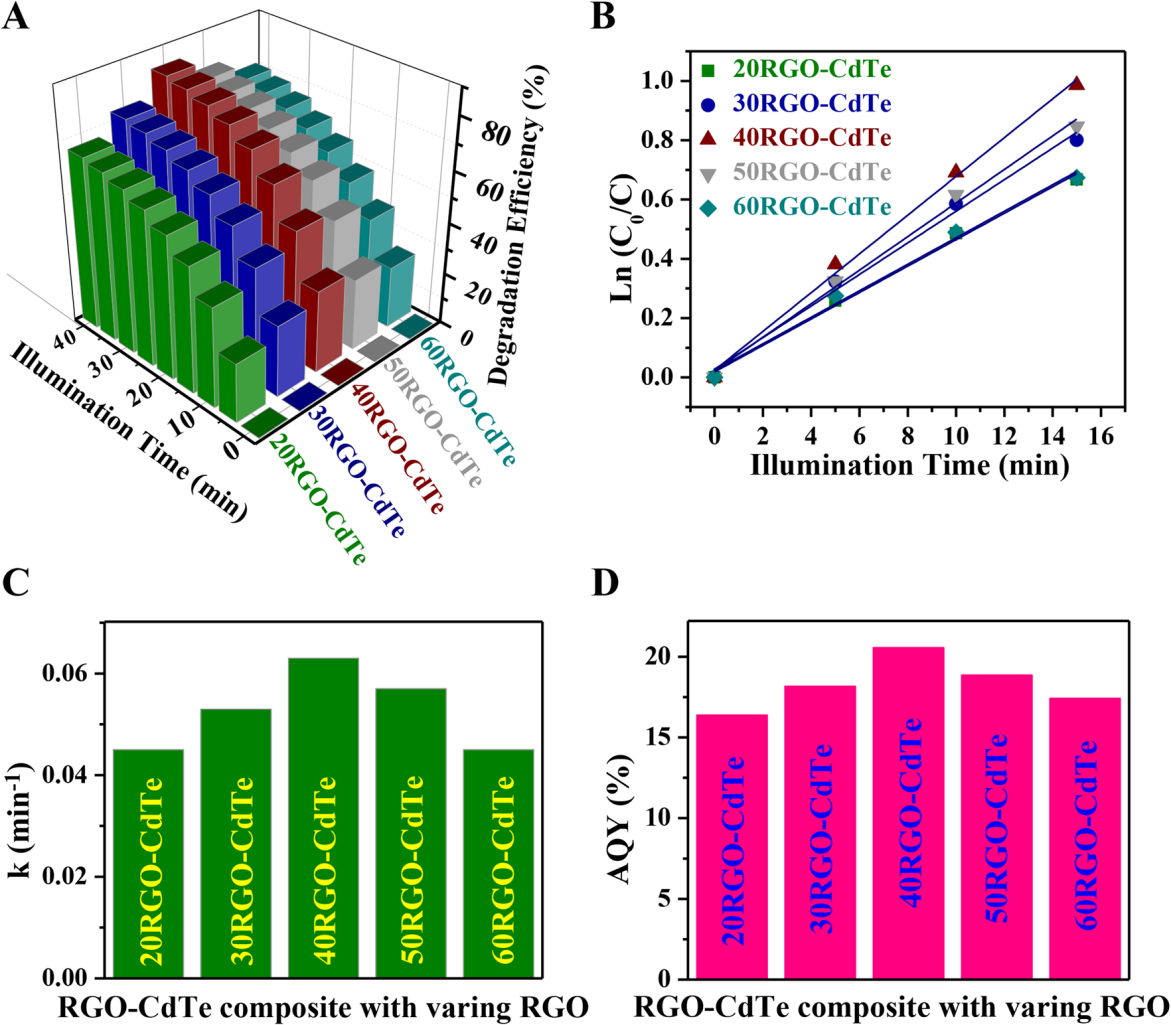
Variation of (**A**) degradation efficiency and (**B**) *Ln (C*_0_/*C)* with illumination time for different amounts of RGO in the RGO-CdTe composite. Comparison of (**C**) *k* and (D) *AQY* for different amounts of RGO in the RGO-CdTe composite.

To establish the RGO-CdTe composite as a potential photocatalyst for the degradation of TC antibiotics, it is obvious to find the ratio of RGO and CdTe in the composite for achieving the highest photocatalytic efficiency. To find the composite with maximum efficiency, the TC degradation was studied with different amounts of RGO in the composite keeping the CdTe amount fixed. Figure S4 in SI presents the absorption spectrum of TC antibiotics in a water solution with varying RGO content in the composite. The highest degradation efficiency was obtained for the 40RGO-CdTe composite, which is mentioned as RGO-CdTe in this report. The variation of degradation efficiency and *Ln*(*C*_0_/*C*) with the time of illumination is presented in Fig. 5A,B respectively. All the composites follow pseudo-first-order kinetics, and the *k* values were estimated from the linear fit. The values of *k* for the composites of RGO-CdTe where RGO:CdTe = 20:1, 30:1, 40:1, 50:1, 60:1 are compared in Fig. 5C, where a maximum *k* is observed for the 40RGO-CdTe composite, which is mentioned as RGO-CdTe in this report. The *AQY* has also been evaluated for different ratios of RGO and CdTe. Like degradation efficiency and *k*, the 40RGO-CdTe composite (RGO-CdTe) shows the highest value of *AQY* (Fig. 5D). The observed highest value at this particular composite might be due to the most efficient photo-induced charge generation under illumination, the key factor of a potential photocatalyst.

**Figure 6:**
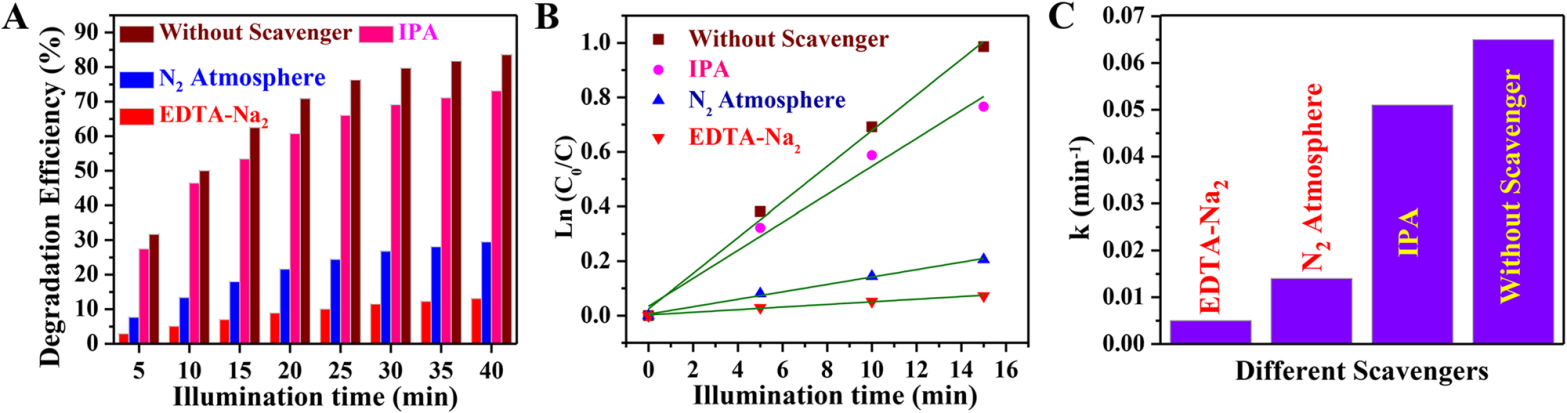
Variation of (**A**) degradation efficiency with the time of illumination and (**B**) *Ln (C*_0_/*C)* with the time of illumination for the RGO-CdTe composite in presence of different scavenger and without scavenger. (**C**) Comparison of *k* for the RGO-CdTe composite in presence of different scavenger and without scavenger.

To explore the reactive species involved in the photocatalytic degradation of TC, scavenger experiments were carried out. Herein, isopropyl alcohol (IPA), N_2_ atmosphere, and ethylenediamine tetraacetic acid disodium salt (EDTA-Na_2_) were selected as the scavengers for the hydroxyl radicals (OH^·^), superoxide anion radical (*O*_2_^*−*·^), and hole (*h*^+^), respectively^35,61–63^. Figure S5 in SI presents the temporal absorption spectral changes of aqueous TC solution containing RGO-CdTe and different quenchers for different reaction species, during the photodegradation process. Figure 6A,B depicts the time variation of degradation efficiency and *Ln*(*C*_0_/*C*), respectively of RGO-CdTe composite for various scavengers of particular sensitive species. From the linear fit of *Ln*(*C*_0_/*C*) versu* t* plot the degradation rate constant of RGO-CdTe for different quenchers was evaluated and is compared in Fig. 6C. It is observed from Fig. 6A that when IPA (OH^·^ quencher) is added, the TC degradation rate decreases slightly, from 83.6% to 73.1%, demonstrating that the OH^·^ has some contribution to TC deterioration. While the photocatalytic activity of RGO-CdTe towards TC degradation is decreased to 29.5% with the purging of N_2_ gas during photocatalysis, establishing that *O*_2_^*−*·^ has also a significant part in degrading TC. The degradation efficiency of the RGO-CdTe composite decreases drastically to 13.8% in the presence of EDTA-Na_2_, demonstrating that the *h*^+^ is the predominant active species for the degradation of TC photo-catalytically, while *O*_2_^*−*·^ and OH^·^ have additionally contributed to the degradation process.

When CdTe is illuminated by solar radiation, excitons are generated, which dissociate into free charge carriers i.e. electrons and holes. In the CdTe, these photo-generated charge carriers have a tendency of recombination which reduces the catalytic performances of CdTe. When CdTe is attached to the RGO mat, the photo-generated electrons transfer easily from the CB of CdTe to the RGO mat, leaving the hole at the valence band (VB) of CdTe due to the favorable band position of CdTe and RGO^64,65^. A platform for the rapid and efficient transfer of photo-induced electrons from the CB of attached CdTe is provided by the numerous well-coupled contacts between the CdTe particles and RGO nano-mats. As a result, the charge recombination rate is significantly reduced by RGO acting as the essential electron carrier by quickly and effectively seizing and passing the electrons produced optically at the VB of the CdTe sensitizer. The high career mobility of RGO mats is advantageous for the collected electrons to reach quickly to the oxygen present on the surface of composite catalyst or dissolve in water, and will produce *O*_2_^*−*·^. These *O*_2_^*−*·^ radicals play a leading role in the degradation of aqueous TC solution. In addition to that, these *O*_2_^*−*·^ may also interact with the *H*^+^ of water, and *e*^*-*^ present in RGO will create HO_2_^*−*^. These HO_2_^−^ will produce H_2_O_2_ after reacting with *H*^+^ of water and the *H*_2_*O*_2_ will further produce OH^·^ after the reaction with *e*^*−*^ present in RGO^19^. These OH^·^ radicals perform a dominant part in the degradation of aqueous TC solution. Conversely, holes present in the valance band of CdTe will directly react with the TC and play a major role in the degradation of TC.

**Figure 7:**
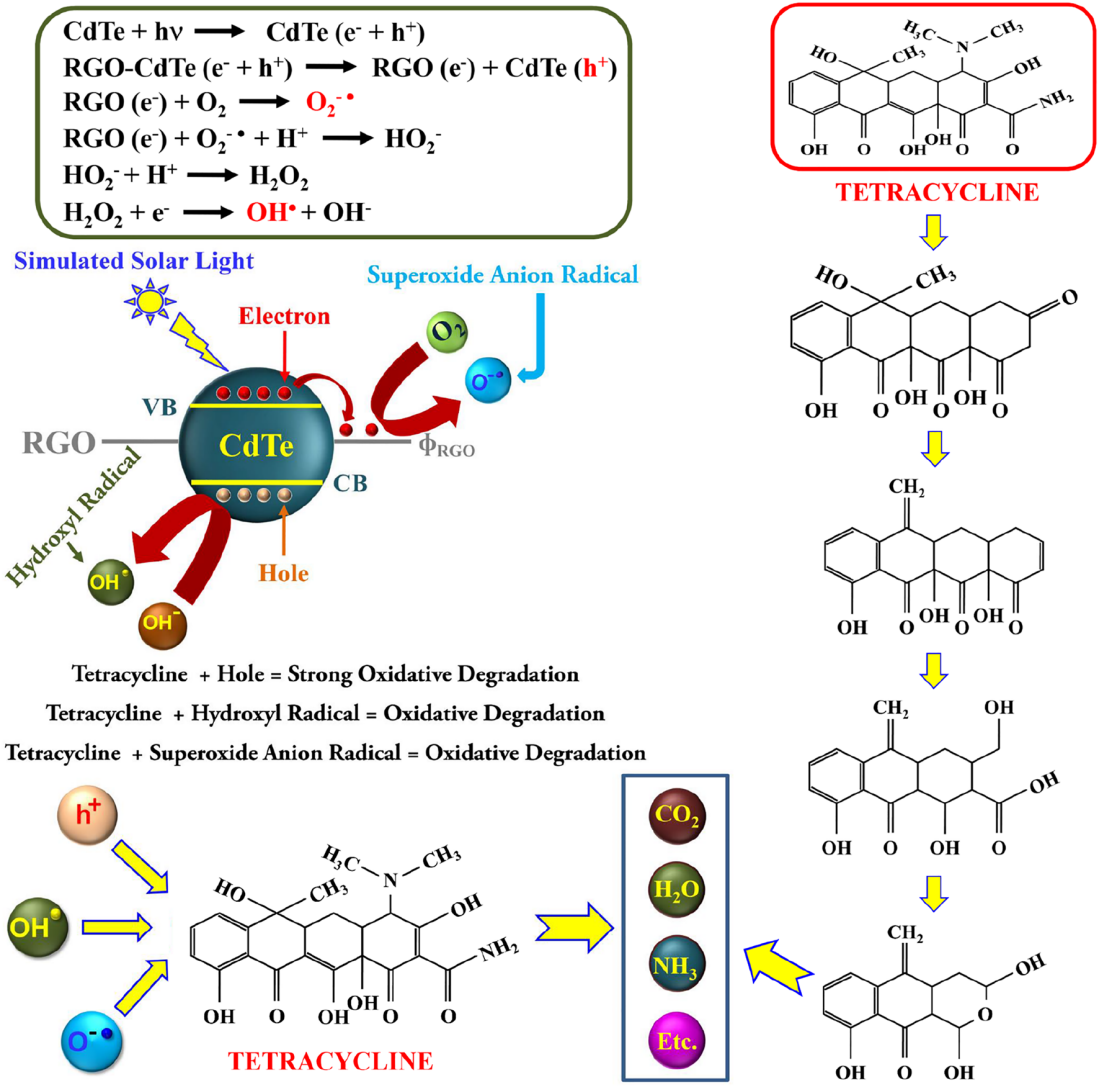
Schematic illustration of TC degradation mechanism by RGO-CdTe composite through photocatalysis process with the mechanism of the formation of the responsive species and the probable TC degradation pathway.

While making a composite of CdTe with RGO, RGO sheets offer a 2D platform for attaching CdTe on it, additionally, the RGO sheets prevent the accumulation of CdTe. In addition to that the wrinkle present in the RGO sheets potentially provides a substantial contact surface between RGO nanosheets and CdTe. In addition to that, after making a composite with RGO the optical absorption of CdTe increases and the PL intensity quenches remarkably. The enhancement of broadband optical absorption and efficient charge transfer without recombination provides better generation of *h*^+^, *O*_2_^*−*·^, and OH^·^, responsible for TC degradation. Based on this knowledge the probable mechanism of photo catalytically degradation of TC using RGO-CdTe composite has been articulated in Fig. 7^66,67^.

The pH-dependent photocatalytic study was performed as the pH of the medium affects the surface charge and electron transfer ability of the photocatalyst. The initial pH of the solution plays an important role in the photocatalytic degradation of TC in an aqueous medium as the surface charge of the TC molecule and the photocatalyst are strongly pH-dependent^68^. At various pH levels, the TC molecule takes on three distinct forms, species that are cationic below pH 3.32, zwitterionic between pH 3.32 and 7.68, and anionic above pH 7.68^68^. To check the effect of pH, we have studied the photocatalytic degradation of TC by RGO-CdTe composite with different pH both acidic and alkaline. 30 mL of TC solution (40 mg/L) and 30 mg of catalyst were added to the photocatalytic reactor, and the pH of the solution system was adjusted to 3 and 5 using sulfuric acid, and that of 9 and 11 with Potassium hydroxide respectively.

**Figure 8:**
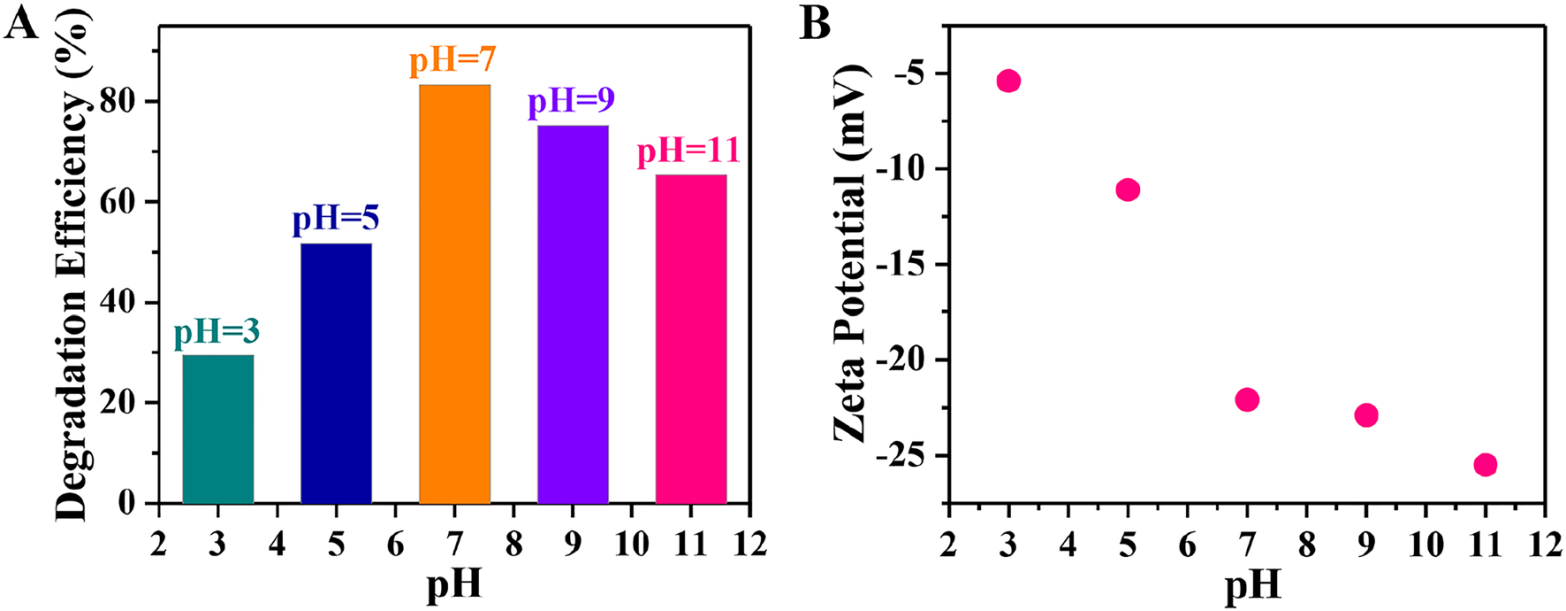
Variation of (**A**) Degradation Efficiency and (**B**) Zeta Potential of RGO-CdTe composite with the pH.

Figure 8A compares the degradation efficiency of the RGO-CdTe composite for different pH. For highly acidic reaction conditions (pH = 3), the photodegradation efficiency was only 29.4%, which increases to 51.8% in a much less acidic environment (pH = 5), which further increases to 83.6% at pH = 7. However, it decreases again to 75.1% and 65.4% for the further increase of pH to 9 and 11 respectively. To get a clear explanation of pH-dependent catalytic performance we have studied the surface properties.

Zeta potential measurements were carried out to look at the surface characteristics of the RGO-CdTe composite at pH 3, 5, 7, 9, and 11 (acidic, neutral, and alkaline) and are presented in Fig. 8B. Zeta potential, which is the electrical potential between the layer of ions bound to the particle surface and the aqueous solution, basically gives the stability of the materials and electrostatic qualities. A negative Zeta Potential was observed in the pH range from 3 to 11. A similar variation of zeta potential was observed for RGO and RGO-based nanomaterials^69,70^. When the photocatalysis was carried out in neutral condition (pH = 7), no repulsive force acted between the negative surface of RGO-CdTe composite and zwitterionic TC molecules. At this pH, the zeta potential of RGO-CdTe is − 22 mV indicating a moderately stable dispersion in the aqueous medium^71^. Stable dispersion always improves the effective surface area of the catalysts to receive a large no of photon particles and as a result, maximum photodegradation efficiency was observed in pH 7. On the other hand, when the pH value was less than neutral (pH = 5 and 3), although there was a chance of an increase in photodegradation efficiency as the attractive force between the negative RGO-CdTe surface and the catatonic TC (for pH = 3) and the absence of any repulsive force between the negative RGO-CdTe surface and the zwitterionic TC (pH = 5). However, in this pH range, the zeta potential of the catalysts is only − 6 and − 11 mV respectively, which indicates an un-stability of colloidal dispersions in the aqueous medium^71^. Thus, there is a possibility of the formation of cluster which reduces the effective surface area to reach TC molecules and hence the photocatalytic efficiency of RGO-CdTe decreases with the decrease of pH below 7. Particles that have zeta potentials more than + 30 mV or less than − 30 mV are generally, thought to produce stable dispersions as a result of interparticle electrostatic repulsion^71^. Thus, at pH 9 and 11 the RGO-CdTe formed a stable dispersion in the aqueous medium which helps to come close to a large number of TC molecules to degrade photo catalytically. At the same time, for these pH values, both the TC molecules and the catalyst surface become negatively charged (anionic forms). The repulsive force between the catalysts and the TC molecules prevents them from approaching each other and subsequently decreases the photocatalytic degradation efficiency^68^. In addition to that the alkaline pH will hinder the formation of one of the reactive species (OH^·^) responsible for the photodegradation of TC^72^.

To check the reusability of the RGO-CdTe composite, a recycling test was carried out. The RGO-CdTe sample was collected by filtration, followed by cleaning with DW and ethanol and dried after every cycle of photocatalytic test during the recycling process. The dried powdered sample was used to degrade a fresh batch of TC. The photocatalytic activity of RGO-CdTe was shown to preserve 90% of its original activity after five successive photodegradation cycles, as illustrated in Fig. S6A in SI. This reduction in photocatalytic performance may be attributed to the adsorption of the residual TC on the photocatalyst surface or the weight loss of RGO-CdTe during each cycle of recycled studies^73^.

The stability of the RGO-CdTe photocatalyst was confirmed by XRD analysis. The XRD pattern of RGO-CdTe after five recycle uses is presented in Figure S6B in SI. The crystalline phase and structure of the RGO-CdTe composite remain unchanged after five recycle-used for the photocatalytic degradation of TC. A comparative analysis of the degradation efficiency for different RGO-based composite photocatalysts reported for TC degradation is presented in Table T1 in SI^19,33–37^.

**Figure 9:**
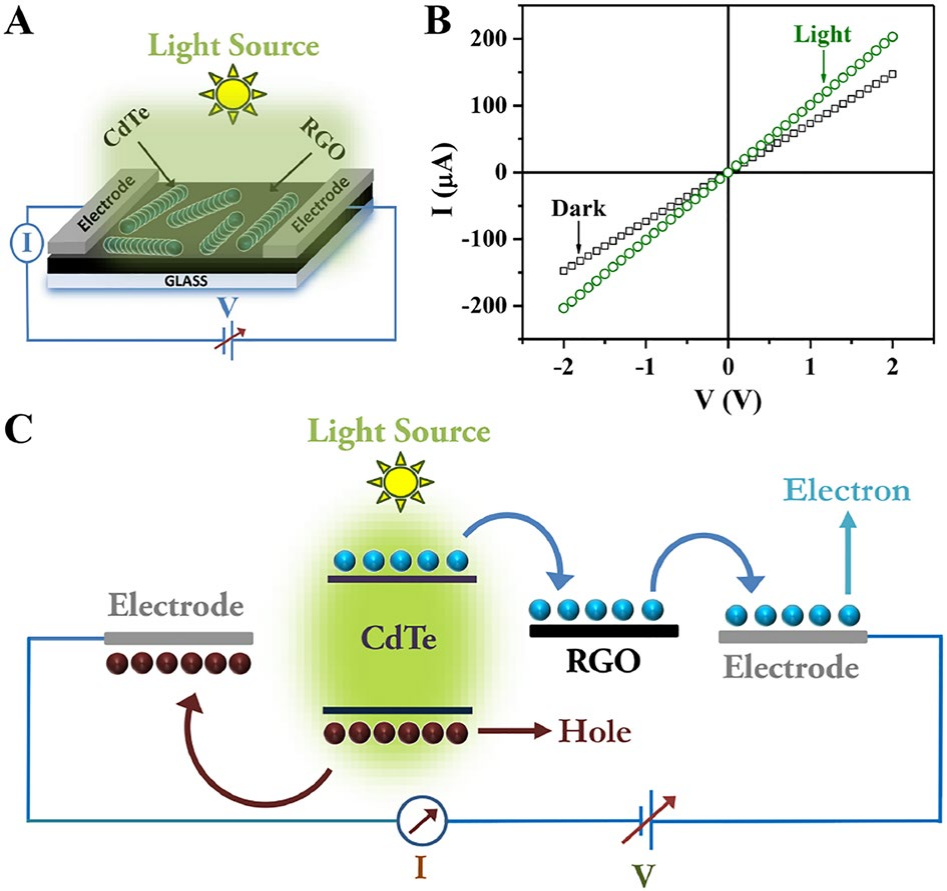
(**A**) Cartoon of the RGO-CdTe thin film photodetector device. (**B**) I-V characteristics of RGO-CdTe thin film under dark and light illumination. (**C**) Mechanism of photocurrent generation in the RGO-CdTe thin film photodetector device.

To establish the photoinduced charge generation in the RGO-CdTe composite under light illumination, we fabricated an RGO-CdTe thin film photodetector and measured the photocurrent generation under solar light illumination. The cartoon of RGO-CdTe thin film photodetector is presented in Fig. 9A. The current-voltage (I-V) characteristics of the thin film device under dark and light illumination are shown in Fig. 9B. Both the curves pass through the origin and show the ohmic variation of current with voltage. An increase in current was observed after the illumination. The mechanism for photocurrent generation in the device is articulated in Fig. 9C. Under illumination, excitons are generated in CdTe which dissociates into free charge carriers, electrons in CB, and holes in VB. Due to the favorable band position of CdTe and RGO, the electrons transfer quickly and efficiently from the CB of CdTe to the attached RGO sheet. The high carrier mobility of the RGO sheet helps the electrons to reach the positive electrons. On the other hand, the holes transfer to the negative electrodes. In this way, the carrier concentration in CdTe increases under light illumination which supports the photocurrent generation in the composite under illumination.

## Conclusion

In conclusion, this work efficaciously developed a potential photocatalyst for the degradation of TC by coupling CdTe with RGO by an easy-to-achieve solution processable hydrothermal method. The RGO-CdTe composite exhibited the degradation efficiency and *k* as 83.6% and 0.062 min^−1^ for the photocatalytic degradation of TC, which is 2.63 and 4.13 times higher than CdTe, respectively. The RGO-CdTe composite showed a high apparent quantum yield of 22.29% for the degradation of TC. Here CdTe acts as a primary photocatalyst where electrons and holes are generated under light illumination. After the incorporation of RGO, the optical absorption of the composite increases notably in the whole visible range (200–900 nm) as well as decreases the probability of electron-hole recombination and subsequently increases the photocatalytic performance. The scavenger investigation demonstrated that although holes are the leading active species, despite that, superoxide and hydroxyl radicals have also dominated roles, in the degradation of TC. The degradation efficiency of RGO-CdTe is strongly pH dependent and shows maximum efficiency at neutral pH and decreases both in the acidic and base medium for the degradation of TC and this property was successfully justified by the zeta potential of RGO-CdTe measured at different pH. The reusability and stability study shows that after using the RGO-CdTe photocatalyst five times, only a 10% performance deterioration occurs. An excellent photocurrent generation in RGO-CdTe thin film device under light illumination has also been observed. The present work demonstrates a promising prospect for using the RGO-CdTe composite as an eco-friendly method for the removal of organic pollutants from aqueous environments. The catalytic performance can be further tuned by improving AQY and the initial pH of the reaction medium.

### Supplementary Information

Supplementary Information.

## Data Availability

The datasets used and/or analyzed during the current study are available from the corresponding author on reasonable request.
