# Supplementary material for: Photocatalytic degradation of tetracycline antibiotics by RGO-CdTe composite with enhanced apparent quantum efficiency
Source: Sci Rep. 2023 Nov 3;13:19028. doi: 10.1038/s41598-023-46120-0 (PMC10624855; doi:10.1038/s41598-023-46120-0)
Supplement: Supplementary file 1 — Supplementary Information. [file 41598_2023_46120_MOESM1_ESM.docx]

### ***Supporting Information for***

### **Photocatalytic Degradation of Tetracycline Antibiotics by RGO-CdTe Composite with Enhanced Apparent Quantum Efficiency**

Suvendu Ghosh^1^, Koushik Chakraborty^1^, Tanusri Pal^2,*^, Surajit Ghosh^1,*^

^1^Department of Physics, Vidyasagar University, Midnapore 721102, WB, India

^2^Department of Physics, Midnapore College, Midnapore 721101, WB, India

^*^Corresponding authors: ^1^surajit@mail.vidyasagar.ac.in (SG);

^2^tanusripal@midnaporecollege.ac.in (TP)


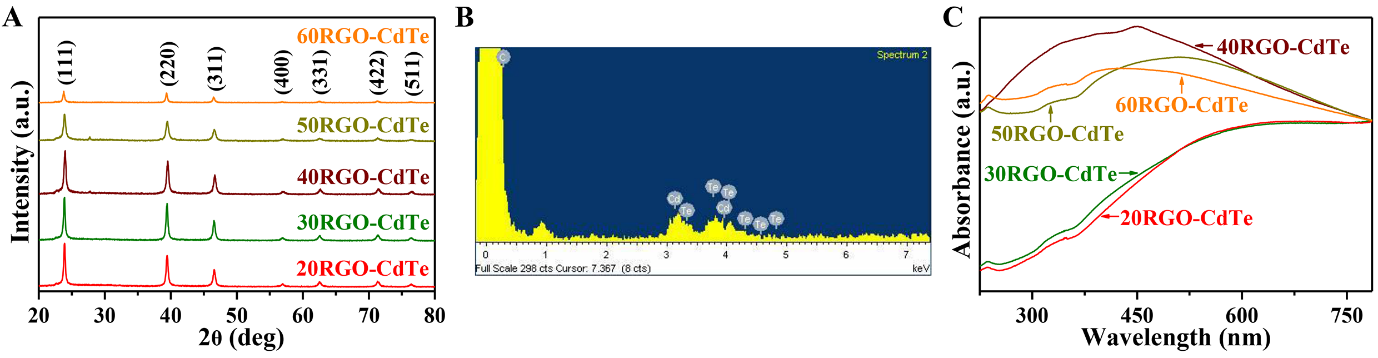


**Figure S1:** (A) XRD pattern of RGO-CdTe composite with varying RGO content in the composite. (B) HRTEM-EDX of RGO-CdTe composite (C) UV-Vis absorption spectra of RGO-CdTe composite with varying RGO content in the composite.


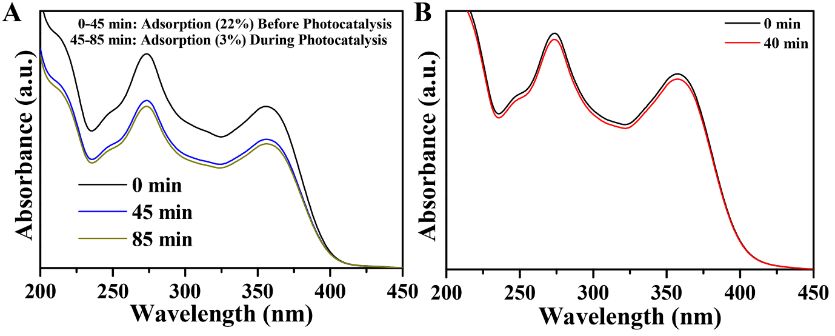


**Figure S2:** Temporal absorption spectral changes of TC during the adsorption process (A) with RGO-CdTe composite in darkness and (B) without RGO-CdTe composite under illumination.


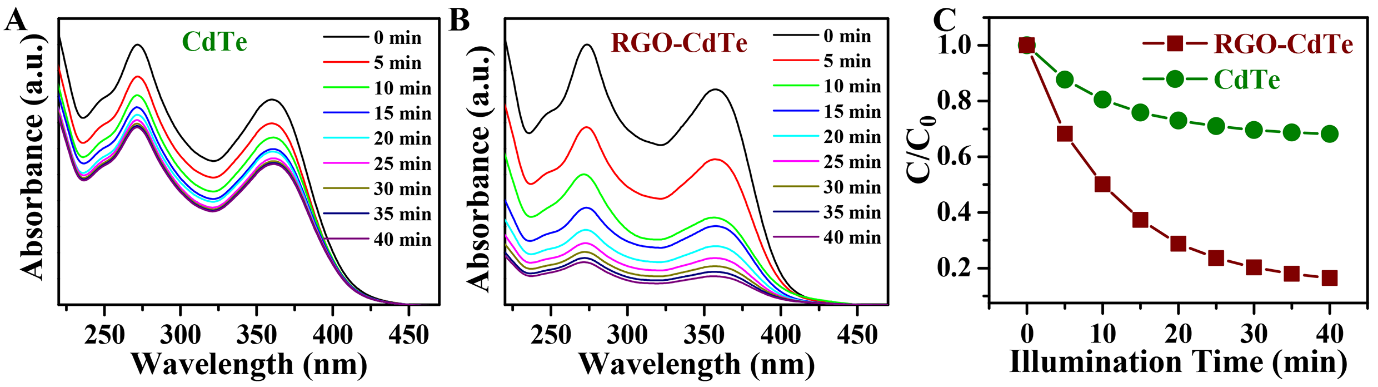


**Figure S3:** Temporal absorption spectral changes of TC during the photodegradation process over (A) CdTe and (B) RGO-CdTe composite (C) Comparison of the temporal changes of TC concentration, as monitored by the UV−vis absorption spectra over illuminated CdTe and RGO-CdTe composite.


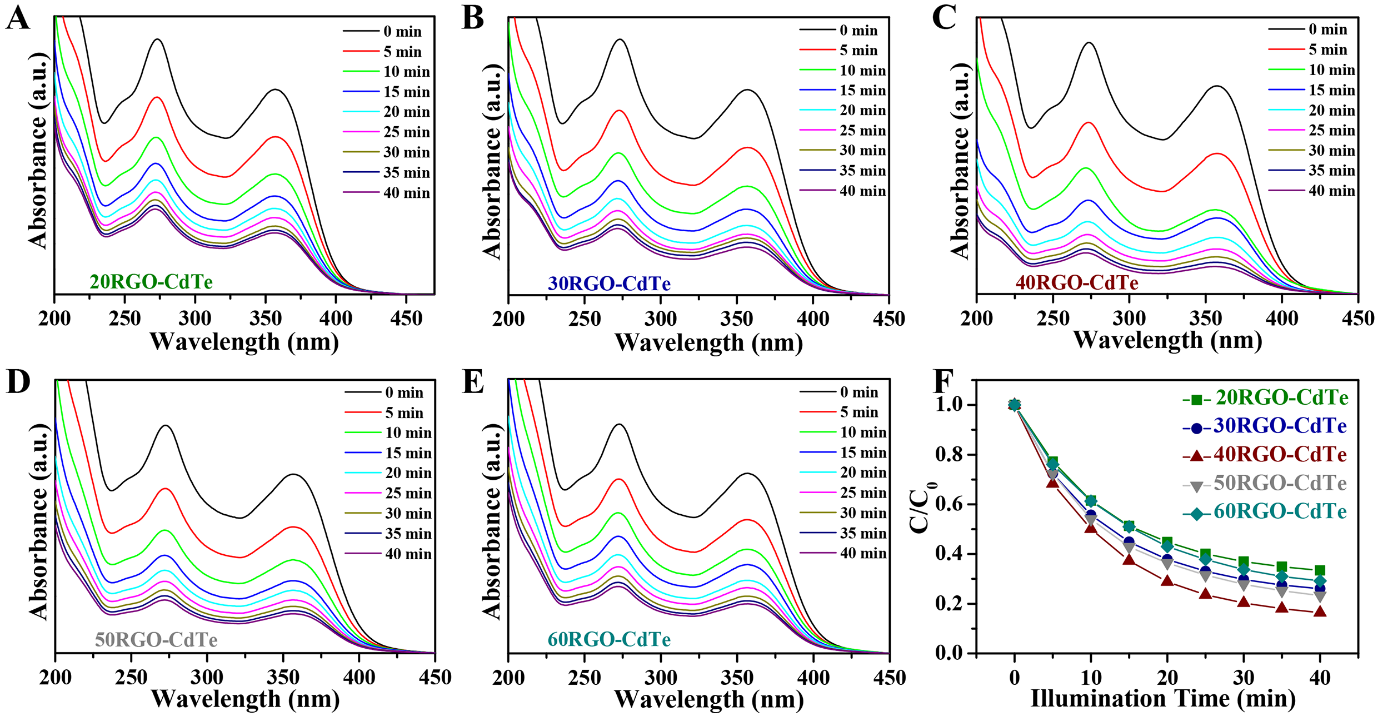


**Figure S4:** Temporal absorption spectral changes of TC during the photodegradation process over (A) 20RGO-CdTe (B) 30RGO-CdTe (C) 40RGO-CdTe (D) 50RGO-CdTe (E) 60RGO-CdTe composite (F) comparison of the temporal changes of TC concentration, as monitored by the UV−vis absorption spectra over illuminated 20RGO-CdTe, 30RGO-CdTe, 40RGO-CdTe, 50RGO-CdTe, and 60RGO-CdTe composite.


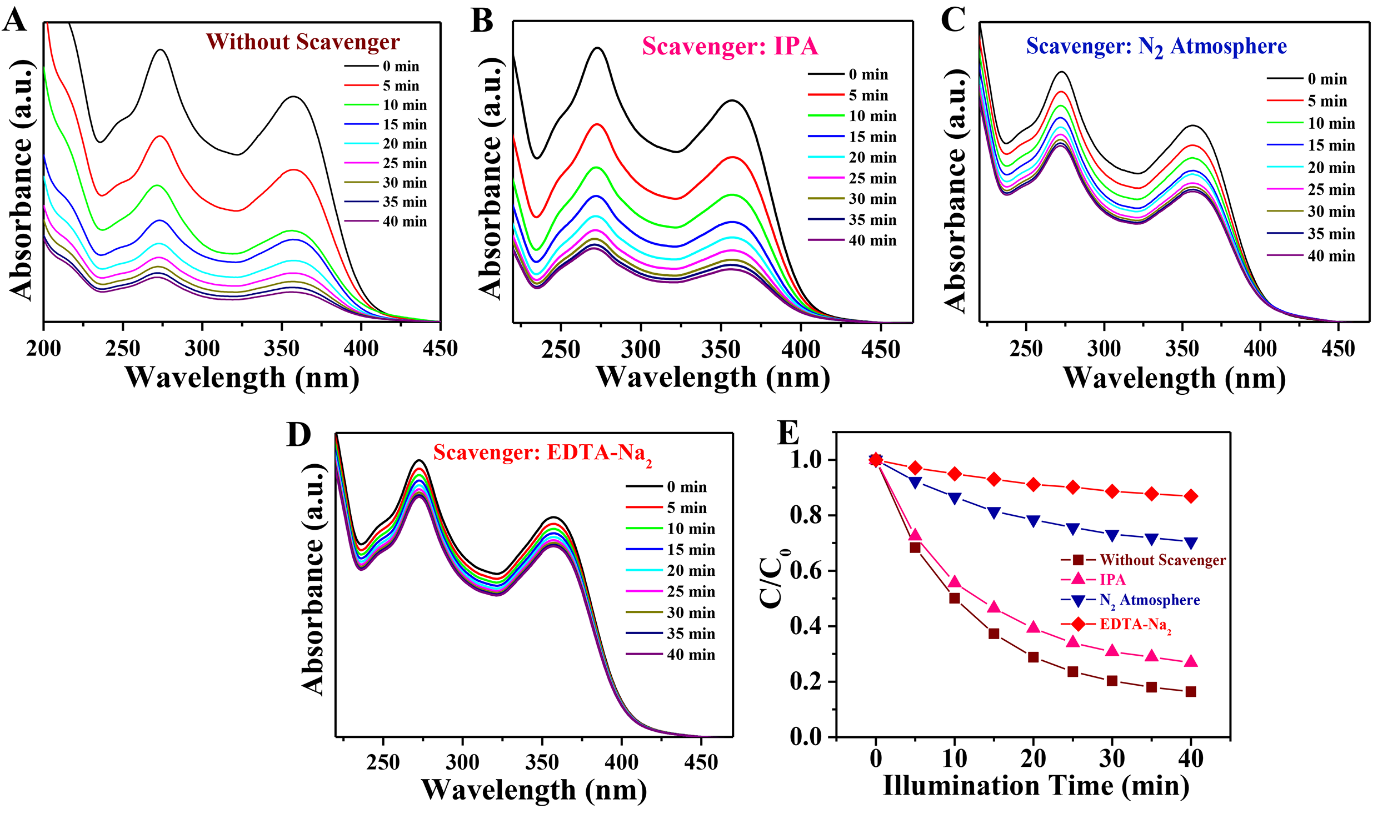


**Figure S5:** Temporal absorption spectral changes of TC during the photodegradation process (A) without any scavenger and with (B) IPA (C) N_2_ Atmosphere and (D) EDTA-Na_2_ scavenger (E) comparison of the temporal changes of TC concentration, as monitored by the UV−vis absorption spectra over illuminated RGO-CdTe composite in absence of any scavenger and in presence of IPA, N_2_ Atmosphere and EDTA-Na_2_ scavenger.


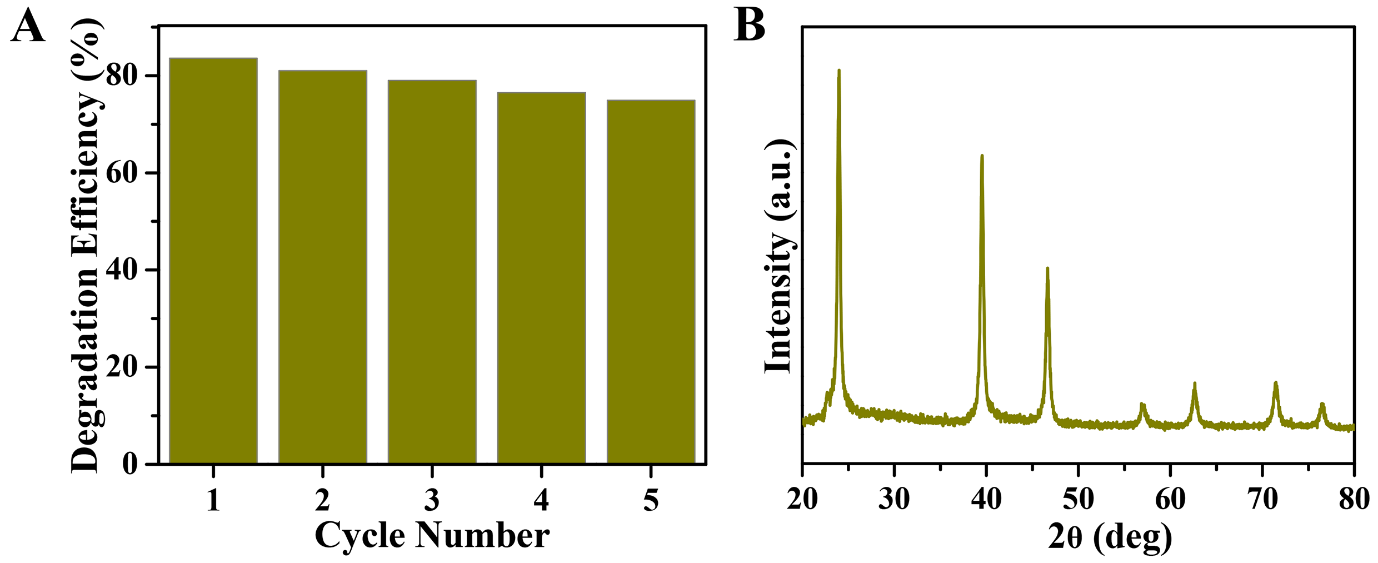


**Figure S6:** (A) Comparison of degradation efficiency for five consecutive cycling runs under visible light illumination (B) p-XRD of RGO-CdTe photocatalyst after five cycles of TC degradation in the aqueous medium.

**Calculation of Apparent Quantum Yield (AQY)**

Wavelength of light λ = 565 nm = 565 × 10^-9^ m

Energy of one photon $E= \frac{hc}{\lambda}=\frac{6.6\times{10}^{-34}\times3\times{10}^{8}}{565 \times10-9}=3.50 \times{10}^{-19} Joules$

The total energy of light falling per second per unit area is

$$E_{Total}=100 mW {cm}^{-2}= 100 \times{10}^{-3}\times{10}^{4}W m^{-2}= 1000 W m^{-2}$$

Number of Photon = $\frac{E_{Total}}{E}= \frac{1000}{3.50 \times{10}^{-19}}=285.7\times{10}^{19}=2.86\times{10}^{21}$

Area of exposed solution =

$$\frac{2\pi rl}{2}= 2\pi rl=3.14\times1.85\times{10}^{-2}\times3.7\times{10}^{-2}=2.15\times{10}^{-3} m^{2}$$

Total number of Photon falling on the solution = $2.86\times{10}^{21}\times2.15\times{10}^{-3}$

= $6.15\times{10}^{18}$

Apparent Quantum Yield (AQY) =$\frac{Number of degraded molecule}{Number of incident photon} \times100$

(AQY)_RGO-CdTe_ = $\frac{1.64\times{10}^{18}\times0.836}{6.15\times{10}^{18}} \times100 \%$ = 22.29%

(AQY) _CdTe_ = $\frac{1.64\times{10}^{18}\times0.318}{6.15\times{10}^{18}} \times100 \%$ = 8.48%

Table T1 The comparison of various synthesized photocatalysts for TC degradation with the current work

| Photocatalyst | Light Source | TC concentration (ppm) | Catalyst dose (gm/L) | Illumination time (min) | Degradation efficiency (%) | Ref |
| --- | --- | --- | --- | --- | --- | --- |
| RGO-Cu_2_O/Bi_2_O | Xenon lamp | 10 | 0.5 | 180 | 75 | 1 |
| La_2_Zr_2_O_7_/rGO | Xenon lamp | 30 | 1 | 40 | 82.1 | 2 |
| rGO-Bi_2_WO_6_ | Xenon lamp | 20 | 0.2 | 60 | 77.3 | 3 |
| RGO-ZnTe | Solar Simulator | 10 | 2 | 45 | 70 | 4 |
| BiVO_4_/FeVO_4_@rGO | Xenon lamp | 30 | 0.6 | 90 | 91.5 | 5 |
| RGO/CdIn_2_S_4_/g-C_3_N_4_ | Xenon lamp | 10 | 1 | 180 | 74 | 6 |
| RGO-CdTe | Solar Simulator | 40 | 1 | 40 | 83.6 | Present work |

**References**

1. Shen, H., Wang, J., Jiang, J., Luo, B., Mao, B. & Sh, W. All-solid-state Z-scheme system of RGO-Cu_2_O/Bi_2_O_3_ for tetracycline degradation under visible-light irradiation, *J. Chem. Eng.* **313**, 508–517. http://dx.doi.org/10.1016/j.cej.2016.11.161 (2017).
2. Wang, Z., Wang, Y., Huang, L., Liu, X., Han, Y. & Wang, L. La_2_Zr_2_O_7_/rGO synthesized by one-step sol-gel method for photocatalytic degradation of tetracycline under visible-light, *J. Chem. Eng.* **384**, 123380. https://doi.org/10.1016/j.cej.2019.123380 (2020).
3. Li, X., Zhang, H., Du, X., Wang, S., Zhang, Q., Li, H. & Ye, F. Efficient visible-light-driven degradation of tetracycline by a 2D/2D rGO-Bi_2_WO_6_ heterostructure, *Environ. Res.* **212**, 113326. https://doi.org/10.1016/j.envres.2022.113326 (2022).
4. Chakraborty, K., Pal, T. & Ghosh, S. RGO-ZnTe: A graphene based composite for tetracycline degradation and their synergistic effect. *ACS Appl. Nano Mater.* **1**, 3137–3144. https://doi.org/10.1021/acsanm.8b00295 (2018).
5. Yang, R., Zhua, Z., Hua, C., Zhong, S., Zhang, L., Liua, B. & Wang, W. One-step preparation (3D/2D/2D) BiVO_4_/FeVO_4_@rGO heterojunction composite photocatalyst for the removal of tetracycline and hexavalent chromium ions in water. *J. Chem. Eng.* **390**, 124522. https://doi.org/10.1016/j.cej.2020.124522 (2020).
6. Xiao, P., Jiang, D., Ju, L., Jing, J. & Chen, M. Construction of RGO/CdIn_2_S_4_/g-C_3_N_4_ ternary hybrid with enhanced photocatalytic activity for the degradation of tetracycline hydrochloride. *Appl. Surf. Sci.* **433**, 388–397. https://doi.org/10.1016/j.apsusc.2017.10.028 (2018).
